# Cetuximab in Kombination mit 5‐Fluorouracil bei Patienten mit fortgeschrittenem kutanem Plattenepithelkarzinom: eine retrospektive Kohortenstudie

**DOI:** 10.1111/ddg.15695_g

**Published:** 2025-08-11

**Authors:** Julia Huynh, Thomas Eigentler, Rose K. C. Moritz, Gabriela Poch, Max Schlaak, Gabor Dobos

**Affiliations:** ^1^ Klinik für Dermatologie Venerologie und Allergologie Charité – Universitätsmedizin Berlin corporate member of Freie Universität Berlin and Humboldt Universität zu Berlin

**Keywords:** 5‐FU, Anti‐EGFR, Cetuximab, Chemotherapie, Kutanes Plattenepithelkarzinom, 5‐FU, anti‐EGFR, chemotherapy, cetuximab, cutaneous squamous cell carcinoma

## Abstract

**Hintergrund:**

Für Patienten mit lokal fortgeschrittenem oder metastasiertem kutanem Plattenepithelkarzinom (lf/m‐PEK), die weder für eine kurative Operation oder Bestrahlung noch für eine Therapie mit PD1‐Inhibitoren infrage kommen, stellt die Kombination aus EGFR‐Inhibitoren und Chemotherapie eine Behandlungsoption dar.

**Patienten und Methodik:**

In dieser monozentrischen, retrospektiven Studie haben wir die Daten von 20 Patienten mit lf/mPEK analysiert. Vier von 20 Patienten hatten ein lfPEK und 16/20 Patienten hatten ein m‐PEK. Die Patienten erhielten zwischen 2015 und 2023 eine Kombination aus Cetuximab und 5‐FU. Neun Patienten erhielten Cetuximab + 5‐FU als Zweitlinientherapie (8 Patienten nach Anti‐PD‐1, 1 Patient nach Radiochemotherapie).

**Ergebnisse:**

Ein Patient hatte eine partielle Remission und 9/20 (45%) hatten eine stabile Erkrankung. Die Krankheitskontrollrate betrug 50%. Es wurden keine kompletten Remissionen beobachtet. Einer der Non‐Responder litt an einem lfPEK, neun Patienten hatten ein m‐PEK mit Fernmetastasen und lokoregionären Lymphknotenmetastasen. Die Therapie war gut verträglich, mit einem medianen progressionsfreien Überleben von 3 Monaten (95%‐KI 2–nicht erreicht) und einem medianen Gesamtüberleben von 29 Monaten (95%‐KI 11–nicht erreicht). Die häufigste Nebenwirkung war ein akneiformes Exanthem.

**Schlussfolgerungen:**

Für Patienten mit fortgeschrittenem PEK, bei denen die Erstlinientherapie mit Cemiplimab kontraindiziert ist oder darunter ein Erkrankungsprogress beobachtet wurde, ist die Kombination von Cetuximab und 5‐FU eine gut verträgliche Behandlungsoption, welche zu einer Krankheitskontrolle führen kann.

## EINLEITUNG

Das kutane Plattenepithelkarzinom (PEK) ist der am zweithäufigsten diagnostizierte Hautkrebs in der hellhäutigen Bevölkerung mit steigender Inzidenz. Chronische UV‐Exposition, fortgeschrittenes Alter und Immunsuppression sind Risikofaktoren für ein kutanes PEK.^1^, ^2^, ^3^

Es sind klinische (Lokalisation, horizontaler Tumordurchmesser und Immunsuppression) und histopathologische (vertikale Tumordicke, Desmoplasie, Differenzierungsgrad, perineurales Wachstum) Risikofaktoren für Metastasierung und das krankheitsspezifische Überleben bekannt.^4^

Die Behandlung des kutanen PEK erfolgt nach den aktuellen europäischen und deutschen Leitlinien.^5^, ^6^ Für Patienten mit metastasiertem und lokal fortgeschrittenem PEK, bei denen eine kurative Operation und/oder Strahlentherapie nicht möglich ist, wird eine Immuntherapie mit den PD1‐Antikörpern Cemiplimab oder Pembrolizumab als systemische Erstlinientherapie empfohlen.^7^ Bei immunsupprimierten Patienten nach einer Organtransplantation besteht ein erhöhtes Risiko für eine Metastasierung. Eine systemische Therapie mit Cemiplimab ist bei ihnen aufgrund des Risikos einer Organabstoßung oder einer Graft‐versus‐Host‐Erkrankung kontraindiziert.^8^ Für diese Patienten und für Patienten, die unter einer Anti‐PD‐1‐Therapie progredient sind, müssen alternative Behandlungsmöglichkeiten in Betracht gezogen werden.

Es liegen Daten zu verschiedenen Mono‐ oder Polychemotherapieprotokollen vor, jedoch waren die Ansprechraten und die Dauer des Ansprechens unbefriedigend.^9^ Polychemotherapien scheinen wirksamer zu sein als Monochemotherapien, aber es werden häufiger Nebenwirkungen beobachtet. Die Toxizität kann bei dem älteren Patientenklientel mit Komorbidität problematisch sein. Die platinbasierte Chemotherapie ist eine Therapieoption für die Behandlung des lokal fortgeschrittenen oder metastasierten PEK.^10^

5‐Fluorouracil (5‐FU)‐basierte Therapieprotokolle scheinen eine Option für Patienten mit fortgeschrittenem PEK zu sein, bei denen eine Anti‐PD‐1‐Therapie kontraindiziert ist oder deren Tumor unter Anti‐PD‐1 progredient ist.

Die intravenöse Gabe von 5‐FU ist eine gängige Therapie des kolorektalen Karzinoms. Die Toxizität ist dosisabhängig, sodass es auch für ältere Patienten in Frage kommt. Topisches 5‐FU wird häufig bei aktinischen Keratosen eingesetzt und erzielt gute Ansprechraten. Auch für 5‐FU bei kutanem PEK gibt es in der Literatur einige Daten.^11^, ^12^, ^13^ Es gibt jedoch keine systemischen Chemotherapien, die für Patienten mit lokal fortgeschrittenem oder metastasiertem PEK zugelassen sind.^6^

Der EGFR‐Inhibitor Cetuximab wird in Kombination mit einer Strahlentherapie oder einer platinbasierten Chemotherapie für das lokal fortgeschrittene oder metastasierte PEK im Kopf‐ und Halsbereich empfohlen. Die Kombination von Cetuximab und einer Strahlentherapie führte zu einer Krankheitskontrollrate von 92% und einer Ansprechrate von 53%. Das progressionsfreie Überleben betrug jedoch nur 5,7 Monate.^14^

Die aktuelle europäischen Leitlinie empfiehlt den EGFR‐Inhibitoren in Kombination mit Chemo‐ oder Strahlentherapie als off‐label Zweitlinientherapie für Patienten mit lokal fortgeschrittenem oder metastasiertem PEK.^6^ Es gibt wenig Daten über die Kombination von Anti‐EGFR und Chemotherapie und unseres Wissens keine Daten über die Kombination von Cetuximab und 5‐FU.

Ziel dieser retrospektiven Studie war es, die Daten von 20 Patienten mit lokal fortgeschrittenem oder metastasiertem PEK, die eine Kombination aus 5‐FU und Cetuximab erhielten, zu analysieren und die Ansprechraten, das Gesamtüberleben und das progressionsfreie Überleben zu bestimmen.

## PATIENTEN UND METHODIK

### Datensammlung

Alle PEK‐Patienten (n = 23), die zwischen April 2015 und Juli 2023 in unserer Klinik 5‐FU und Cetuximab erhielten, wurden in diese Studie eingeschlossen. Zwei Patienten mussten aufgrund unvollständiger Daten ausgeschlossen werden. Ein Patient mit PEK an der Schleimhaut wurde ebenfalls ausgeschlossen. Bei 3/20 Patienten war die Therapie zum Zeitpunkt der Datenextraktion noch nicht abgeschlossen. Die Therapie richtete sich nach den europäischen und deutschen PEK‐Leitlinien unter Verwendung des AJCC‐Staging‐Systems für das kutane PEK, welches zuletzt 2017 aktualisiert wurde, behandelt.^5^, ^6^, ^15^

Die klinischen Daten zu Geschlecht, Alter, Datum der Diagnose, Tumormerkmalen, vorherigen und nachfolgenden Therapien, Angaben zur Tumorprogression und das Todesdatum wurden mittels Durchsicht der elektronischen Patientenakten erhoben. Wurde mehr als ein PEK diagnostiziert, wurde für die Tumoreigenschaften der Tumor mit dem höchsten Risiko für eine Progression nach Brantsch et al. verwendet.^4^ Ob ein Patient der lokal fortgeschrittenen oder der metastasierten PEK‐Gruppe angehörte wurde mit dem Tumorstadium zu Beginn der 5‐FU/Cetuximab‐Therapie festgelegt. Die Klassifikation unerwünschter Ereignisse erfolgte nach den *Common Terminology Criteria for Adverse Events* (CTCAE).^16^

Der primäre Endpunkt war das Gesamtüberleben, definiert als die Zeit vom ersten Therapiezyklus bis zum Tod (durch PEK oder andere Ursachen) oder bis zum Datum der letzten Nachuntersuchung in unserer Klinik. Die Dauer des Ansprechens wurde in Monaten vom Beginn der 5‐FU/Cetuximab‐Therapie bis zur Tumorprogression gemessen.

Ein Votum der Ethikkommission der Charité Universitätsmedizin Berlin lag vor (Referenznummer: EA1/262/22).

### Therapie

Die kombinierte 5‐FU‐ und Cetuximab‐Therapie wurde im stationären Setting verabreicht. Cetuximab wurde in einer Dosis von 400 mg/m^2^ intravenös (i.v.) über 120 Minuten an Tag 1 und danach wöchentlich 250 mg/m^2^ i.v. über 60 Minuten verabreicht. 5‐FU wurde in einer Dosis von 1000 mg/m^2^ über 24 Stunden mit einer Elastomerpumpe von Baxter an Tag 1 und 2 über 24 Stunden alle 3 Wochen verabreicht. Zur Spülung des Infusionssystems wurde physiologische Kochsalzlösung verwendet. Die Patienten wurden engmaschig auf das Risiko einer anaphylaktischen Reaktion überwacht. Ein Dihydropyrimidin‐Dehydrogenase (DPD)‐Mangel wurde vor der Behandlung mit 5‐FU ausgeschlossen.

Außerdem wurden die Haut und die Mundhöhle täglich auf Haut‐ beziehungsweise Schleimhautveränderungen untersucht und das Körpergewicht wurde regelmäßig kontrolliert. Die Patienten wurden prophylaktisch mit topischem Amphotericin B behandelt, um eine Mukositis zu verhindern. Die Prämedikation ist in Tabelle 1 aufgeführt.

**TABELLE 1:** Prämedikation zu jedem Therapiezyklus.

Dexamethason 4 mg in 100 mL Kochsalzlösung	Über 15 min. i.v.
Dimetinden 4 mg	Über 15 min. i.v.
Ondansetron 4 mg	Über 15 min. i.v.
Ondansetron 8 mg	p.o.

Während der Therapie wurden das Differentialblutbild sowie die Leber‐ und Nierenfunktion kontrolliert.

### Statistik

Die Statistik wurde mit SPSS Version 27 (SPSS Inc., Chicago, IL, USA) durchgeführt. Die primären Endpunkte waren das Gesamtüberleben und das progressionsfreie Überleben. Das Gesamtüberleben wurde definiert als die Zeit in Monaten vom ersten Zyklus von Cetuximab/5‐FU bis zum Tod (durch PEK oder andere Ursachen). Das progressionsfreie Überleben war definiert als die Zeit in Monaten vom ersten Therapiezyklus bis zum Progress oder Tod. Alle Ereignisse außer einem Progress wurden zensiert. Wurde kein Ereignis registriert, wurde das Überleben zum Zeitpunkt der letzten Nachuntersuchung zensiert. Die Überlebenswahrscheinlichkeiten wurden nach der Kaplan‐Meier‐Methode geschätzt. Zur Berechnung der medianen Nachbeobachtungszeit wurde die reverse Kaplan‐Meier‐Methode verwendet. Der sekundäre Endpunkt war die Toxizität.

## ERGEBNISSE

### Patientenkohorte

Zwanzig Patienten wurden in die Studie eingeschlossen. Alle Patienten waren männlich. Das mediane Alter bei der Erstdiagnose betrug 74 Jahre (Bereich: 50–83). Die Hälfte der Patienten (50%) war immunsupprimiert, davon waren fünf Patienten nierentransplantiert. Bei drei Patienten wurde eine chronische lymphatische Leukämie und bei einem Patienten eine Osteomyelofibrose diagnostiziert. Ein Patient hatte eine Granulomatose mit Polyangiitis und wurde mit Azathioprin behandelt.

Bei elf Patienten (55%) befand sich der Primärtumor im chronisch UV‐exponierten Kopf‐Hals‐Bereich. Bei drei Patienten war das Stadium bei der Erstdiagnose unbekannt. Bei den meisten Patienten (45%) lag ein AJCC‐Stadium III vor.

Zu Beginn der Therapie mit 5‐FU und Cetuximab hatten vier Patienten (20%) ein lokal fortgeschrittenes PEK und 16 Patienten (80%) ein metastasiertes PEK. Lokoregionale Lymphknotenmetastasen waren bei 16 Patienten (80%) vorhanden. Fernmetastasen wurden bei 14 Patienten (70%) detektiert. Davon hatten zwei Patienten lymphogene Fernmetastasen, ein Patient kutane Fernmetastasen, sechs Patienten Lungenmetastasen, sechs Patienten Parotismetastasen, einer ossäre Metastasen, ein Patient Lebermetastasen und ein Patient Milzmetastasen.

Neun Patienten (45%) hatten zuvor eine systemische Therapie erhalten. Ein Patient hatte zuvor eine Chemo‐ und Strahlentherapie erhalten. Acht Patienten hatten zuvor eine Therapie mit Checkpoint‐Inhibitoren erhalten, sieben davon eine Monotherapie mit Cemiplimab und ein Patient mit Cemiplimab und Pembrolizumab. Bei allen neun Patienten mussten die vorangegangenen Therapien aufgrund eines Tumorprogresses abgesetzt werden.

In der Gruppe der acht Patienten, die 5‐FU und Cetuximab nach Progress unter Anti‐PD‐1 erhielten, hatten fünf Patienten eine stabile Erkrankung und ein Patient ein partielles Ansprechen. Zwei Patienten hatten einen Progress. Bei beiden Patienten lag keine hämatoonkologische Grunderkrankung vor. Das PEK befand sich bei einem Patienten im Kopf‐ und Halsbereich und bei dem anderen Patienten mit Progress an der unteren Extremität.

Bei vier Patienten aus unserer Kohorte war eine Therapie mit Anti‐PD‐1 aufgrund des Zustands nach einer Organtransplantation kontraindiziert. Drei von ihnen hatten eine stabile Erkrankung, einer hatte einen Tumorprogress. Alle transplantierten Patienten waren in einem Zustand nach einer Nierentransplantation. Die Immunsuppression erfolgte mit Mycophenolatmofetil, Tacrolimus und (Methyl‐)Prednisolon sowohl bei dem Patienten mit Tumorprogress als auch bei zwei Patienten mit stabiler Erkrankung. Der Zeitraum zwischen der Organtransplantation und dem Beginn der Therapie betrug bei dem Patienten mit Progress < 10 Jahre und bei den Patienten mit stabiler Erkrankung > 10 Jahre.

Die Patientencharakteristika sind in Tabelle 2 dargestellt.

**TABELLE 2:** Patienten‐ und Tumorcharakteristika.

		n = 20 (%)
Lokalisation des Primarius	Kopf‐/Hals‐Bereich	11 (55%)
	Extremitäten	3 (15%)
	Stamm	5 (25%)
	Okkult	1 (5%)
AJCC‐Stadium bei Erstdiagnose^*^	Stadium I	5 (25%)
	Stadium II	2 (10%)
	Stadium III	9 (45%)
	Stadium IV	1 (5%)
Vortherapien	OP und/oder Radiotherapie	19 (95%)
	Chemotherapie	1 (5%)
	Checkpointinhibition	8 (40%)
Immunsuppression		10 (50%)

* Nicht bekannt bei drei Patienten.

### Wirksamkeit der Therapie mit Cetuximab und 5‐FU

Alle Patienten wurden mindestens über eine Woche behandelt, 9/20 Patienten wurden 12 Wochen oder länger behandelt. Die mediane Dauer der Behandlung betrug 9 Wochen (min. 1 Woche, max. 77 Wochen). Ein Patient zeigte ein partielles Ansprechen. Eine stabile Erkrankung wurde bei 9/20 (45%) Patienten erreicht. Eine stabile Erkrankung wurde bei drei von vier Patienten mit lokal fortgeschrittenem PEK und sechs Patienten mit metastasiertem PEK beobachtet. Vier Patienten hatten Fernmetastasen (einschließlich der Parotis) und lokoregionäre Lymphknotenmetastasen und zwei Patienten hatten ausschließlich lokoregionäre Metastasen. Die Gesamtansprechrate lag bei 5% und die Krankheitskontrollrate bei 50%.

Zehn Patienten (50%) hatten einen Tumorprogress. Ein Patient hatte ein lokal fortgeschrittenes PEK, neun Patienten hatten ein metastasiertes PEK mit lokoregionalen Lymphknoten‐ und Fernmetastasen (einschließlich Parotis). Eine Zusammenfassung der Daten ist in Tabelle 3 dargestellt.

**TABELLE 3:** Therapie von 20 Patienten mit fortgeschrittenem kutanem PEK mit Cetuximab und 5‐FU.

Pat. Nr.	La/m PEK	Immunsuppression	Vortherapien	Zyklen Cetuximab/5‐FU	Ansprechen	Progressions‐freie Zeit in Monaten	Follow up in Monaten
1	mPEK	–	Cemiplimab	43/16	SD	–	12
2	mPEK	NTX	Cemiplimab	47/17	SD	–	12
3	mPEK	NTX	Keine	10/3	SD	–	2
4	lfPEK	Hem	Cemiplimab/Pembrolizumab	36/2	SD	–	4
5	lfPEK	NTX	Keine	14/6	SD	–	17
6	lfPEK	AZA	Keine	7/3	PD	1	1
7	lfPEK	NTX	Keine	2/1	SD	–	2
8	mPEK	–	Keine	12/4	PD	3	11
9	mPEK	–	Keine	9/3	PD	1	3
10	mPEK	CLL	Keine	8/3	SD	–	7
11	mPEK	–	Cisplatin + Radiotherapie	6/2	PD	2	53
12	mPEK	–	Keine	5/2	PD	3	3
13	mPEK	–	Keine	7/3	PD	1	47
14	mPEK	–	Keine	11/2	PD	1	44
15	mPEK	NTX	Keine	9/3	PD	3	29
16	mPEK	–	Cemiplimab	16/6	PD	2	8
17	mPEK	–	Cemiplimab	7/3	PD	2	4
18	mPEK	CLL	Cemiplimab	10/4	SD	–	1
19	mPEK	–	Cemiplimab	15/5	PR	–	3
20	mPEK	CLL	Cemiplimab	15/5	SD	–	3

*Abk*.: Pat, Patient; mPEK, metastasiertes PEK; lfPEK, lokal fortgeschrittenes PEK; NTX, Nierentransplantation; Hem, hämatoonkologische Erkrankung; CCL, chronische lymphatische Leukämie; AZA, Azathioprin; SD, stabile Erkrankung; PD, progressive Erkrankung; PR, partielles Ansprechen

Es gab insgesamt sieben Todesfälle (aufgrund von PEK oder Tod aus anderen Gründen), keiner davon war auf Nebenwirkungen der Therapie mit 5‐FU und Cetuximab zurückzuführen.

**ABBILDUNG 1:**
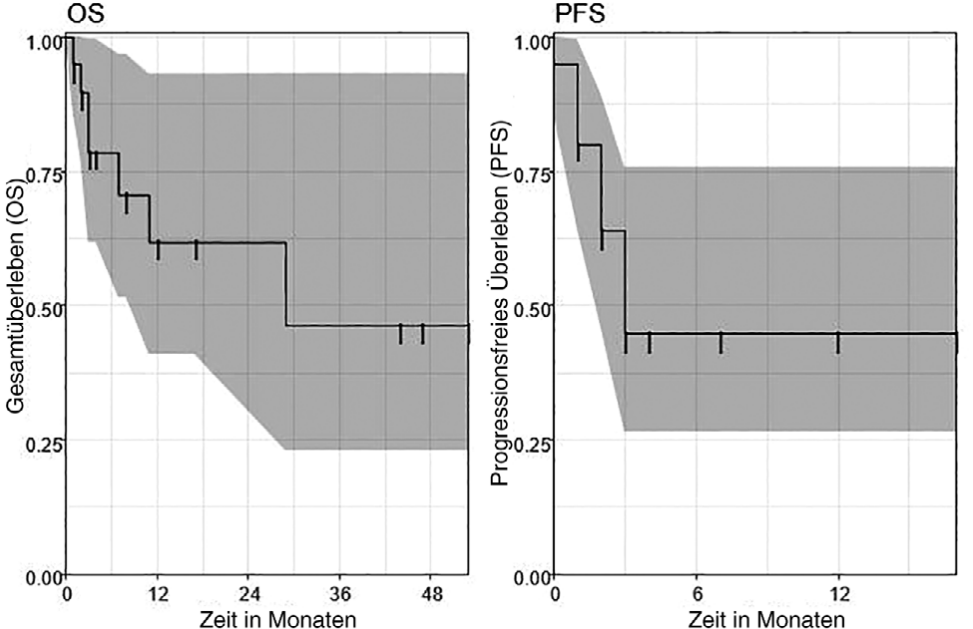
Gesamtüberleben (OS) und progressionsfreies Überleben (PFS) in Monaten entsprechend dem Gesamtkollektiv der Patienten mit kombiniertem Cetuximab und 5‐FU bei fortgeschrittenem cSCC.

Das mediane Gesamtüberleben betrug 29 Monate (95%‐Konfidenzintervall [KI] 11–nicht erreicht). Die Gesamtüberlebensrate lag nach 6 Monaten bei 79% (95%‐KI 62%–100%) und nach 12 und 24 Monaten bei 62% (95%‐KI 41%–93%). Das mediane progressionsfreie Überleben betrug drei Monate (95%‐KI 2–nicht erreicht). Die progressionsfreie Überlebensrate nach 6 und nach 12 Monaten betrug 45% (95%‐KI 26%–76%) (Abbildung 1).

Die mediane Nachbeobachtungszeit betrug 12 Monate (95%‐KI 4–nicht erreicht).

### Nebenwirkungen

Die häufigste dokumentierte Nebenwirkung war ein akneiformes Exanthem (8/20, 40% der Patienten). Bei fünf Patienten traten Hautnebenwirkungen vom CTCAE‐Grad 1–2 auf. Bei drei Patienten trat eine Hauttoxizität des Grades 3–4 auf, sodass eine systemische Behandlung mit Doxycyclin oder Isotretinoin zusätzlich zur topischen Behandlung indiziert war.

Bei einem Patienten der Kohorte wurde eine Myelosuppression (CTCAE‐Grad 3) dokumentiert, die zu einem Behandlungsabbruch führte, da 5‐FU als Ursache nicht ausgeschlossen werden konnte. Es wurden keine weiteren unerwünschten Ereignisse dokumentiert.

## DISKUSSION

Dies ist die erste Studie zum Einsatz der Kombination von 5‐FU und Cetuximab bei Patienten mit fortgeschrittenem kutanen PEK. Wir stellen unsere Ergebnisse zur Wirksamkeit und Toxizität vor und vergleichen sie mit anderen Behandlungsoptionen. Die Kombination ist eine Alternative für Patienten, bei denen eine Erstlinienbehandlung mit Cemiplimab kontraindiziert ist, wie zum Beispiel bei immunsupprimierten Patienten oder Patienten mit Progression unter Anti‐PD‐1, die einen erheblichen Anteil der Patienten mit fortgeschrittenem PEK ausmachen. Die Behandlungsmöglichkeiten für dieses Patientenklientel sind sehr begrenzt.

Das mediane Alter bei Erstdiagnose lag in unserer Patientenkohorte bei 74 Jahren (Bereich: 50–83). Damit handelt es sich um eine überwiegend ältere Kohorte von Patienten mit PEK. Die Hälfte unserer Patienten war immunsupprimiert. Davon waren 50% nierentransplantiert. Bei drei Patienten wurde eine chronische lymphatische Leukämie diagnostiziert, und ein Patient hatte eine Myelofibrose, die mit Ruxolitinib behandelt wurde. Eine Granulomatose mit Polyangiitis lag bei einem Patienten vor. Diese wurde mit Azathioprin behandelt. Neben einer hohen kumulativen UV‐Exposition und einem hellen Hauttyp ist eine Immunsuppression ein Risikofaktor für die Entwicklung von nichtmelanozytärem Hautkrebs.^17^, ^18^ Eine vorliegende Immunsuppression ist auch ein prognostischer Faktor für Metastasierung und das krankheitsspezifische Überleben. Weitere prognostische Faktoren sind: vertikale Tumordicke, Desmoplasie, Differenzierungsgrad, perineurales Wachstum (histopathologische Risikofaktoren), Lokalisation und horizontaler Tumordurchmesser (klinische Risikofaktoren).^15^ Die systemische Behandlung bei immunsupprimierten Patienten stellt eine Herausforderung dar, da die Erstlinienbehandlung mit Cemiplimab zu schweren Komplikationen wie einer Graft‐versus‐Host‐Reaktion oder einer Organabstoßung führen kann.^8^ Für Patienten mit einem Tumorprogress unter der Therapie mit Cemiplimab oder mit Kontraindikationen ist die Kombination aus 5‐FU und Cetuximab eine Off‐Label‐Therapieoption.^6^

Wir konnten keine klinischen Parameter identifizieren, die das Ansprechen auf Cetuximab und 5‐FU bei den acht Patienten vorhersagen konnten, die unter Anti‐PD‐1 progredient waren. Hämatoonkologische Diagnosen oder eine Lokalisation des Tumors in nicht chronisch sonnenexponierten Arealen waren bei den Patienten mit progredienter Erkrankung nicht häufiger vorliegend als bei den Patienten mit stabiler Erkrankung.

In unserer Patientengruppe, welche die Kombination als Erstlinienbehandlung aufgrund des Zustands nach Organtransplantation erhielt, konnten wir ebenfalls keine klinischen Parameter für das Ansprechen auf die Therapie bestimmen. Die immunsuppressive Medikation bei den Patienten mit Progress unterschied sich nicht von den anderen Patienten mit stabiler Erkrankung. Die Zeit zwischen der Transplantation/dem Beginn der Immunsuppression und dem Beginn der Therapie war bei den Patienten mit Progress kürzer als bei den Patienten mit stabiler Erkrankung.

Der PD1‐Antikörper Cemiplimab wird in der aktuellen europäischen Leitlinie als Erstlinientherapie für Patienten mit fortgeschrittenem PEK empfohlen. Eine kombinierte Therapie mit Anti‐EGFR‐ und Radio‐/Chemotherapie wird für Patienten mit Kontraindikationen für eine Immuntherapie empfohlen.^6^ Es liegen keine randomisierten prospektiven Studien zum Vergleich von zielgerichteten Therapien und Chemotherapie bei Patienten mit kutanem PEK vor. Nach unserem Wissen gibt es außerdem keine retrospektiven oder prospektiven Daten in der Literatur über die Ansprechraten auf die Kombination von 5‐FU und Cetuximab. In unserer Studie lag die Ansprechrate bei 5%. Einer unserer Patienten hatte ein partielles Ansprechen und 9/20 Patienten hatten eine stabile Erkrankung unter der Therapie.

Die Krankheitskontrollrate lag bei 50%. Das mediane Gesamtüberleben betrug 29 Monate (95%‐KI 11–nicht erreicht) und das mediane progressionsfreie Überleben betrug 3  Monate (95%‐KI 2–nicht erreicht). Cetuximab und 5‐FU war bei 45% unserer Patienten als Zweitlinientherapie nach PD‐1‐ oder Radiochemotherapie eingesetzt worden.

In der Literatur variieren die Daten zum Ansprechen auf Mono‐ und Polychemotherapieschemata. Die Ansprechraten der Chemotherapie bei fortgeschrittenem PEK liegen in Phase‐II‐Studien in der Literatur zwischen 17% und 84%.^10^, ^19^, ^20^, ^21^ Kramb et al. zeigten in ihrem Patientenkollektiv eine Gesamtansprechrate von nur 17,4%.^21^ Guthrie et al. zeigten bei 28 Patienten, die mit einer Chemotherapie auf Cisplatinbasis behandelt wurden, ein vollständiges Ansprechen bei 28% und ein teilweises Ansprechen bei 40%. Die mediane Dauer des Ansprechens betrug 15 Monate.^10^

Der Antimetabolit 5‐FU, ein Pyrimidinanalogon, wird als Monotherapie oder in Kombinationen zur Behandlung von Magen‐Darm‐, Brust‐ sowie Kopf‐ und Halstumoren eingesetzt. Capecitabin, sein orales Prodrug, ist für die Behandlung von Brust‐, Magen‐ und Dickdarmkrebs zugelassen.^13^, ^15^, ^22^ Cartei et al. zeigten in ihrer Kohorte von Patienten mit kutanem PEK, die mit oralem 5‐FU behandelt wurden, eine Verbesserung bei 9/14 Patienten mit einer medianen Dauer von mehr als 30 Wochen.^11^ Das Ansprechen auf Chemotherapien ist oft begrenzt mit einem kurzen Gesamtüberleben und einem kurzen progressionsfreien Überleben. Die Toxizität kann limitierend sein, da Patienten mit PEK oft älter sind und keine nebenwirkungsreichen Therapien erhalten können.

Montaudie et al. beschrieben eine Ansprechrate von 53% und eine Krankheitskontrollrate von 87% bei PEK‐Patienten, die mit Cetuximab behandelt wurden. In dieser Studie wurde Cetuximab bei den meisten Patienten als Erstlinientherapie eingesetzt, somit sind die Daten mit unserer Patientenkohorte nicht vergleichbar.^23^ Hillen et al. berichteten über eine Ansprechrate von 20% und Maubec et al. über eine Ansprechrate von 28% und eine Krankheitskontrollrate von 69% in ihrer Phase‐II‐Studie zur Erstlinienbehandlung mit Cetuximab.^24^, ^25^

In der Studie von Kamb et al. betrug die Gesamtansprechrate auf eine Kombination aus Cetuximab und Chemotherapie 14,3% bei 14 Patienten. Das mediane progressionsfreie Überleben aller Patienten (jede Therapie) in dieser retrospektiven Studie betrug 15 Wochen, was mit unseren Ergebnissen vergleichbar ist.^21^ Casassa et al. zeigten in ihrer retrospektiven Analyse von 14 Patienten mit PEK, die mit Cetuximab und Paclitaxel behandelt wurden, ein medianes progressionsfreies Überleben von 6 Monaten, aber die Therapie war nebenwirkungsreicher als bei unserer Studie.^26^ Gold et al. zeigten in einer Phase‐II‐Studie mit 29 Patienten mit kutanem PEK, die mit dem EGFR‐Inhibitor Erlotinib behandelt wurden, eine Gesamtansprechrate von 10% und eine Krankheitskontrollrate von 72%. Vorab erfolgten Bestrahlung, Operation und/oder Chemotherapie.^27^

In der Keynote‐629‐Studie wurde bei 105 Patienten, die mit dem PD‐1‐Antikörper Pembrolizumab behandelt wurden, eine Gesamtansprechrate von 34,3% und eine Krankheitskontrollrate von 52,4% ermittelt, wobei 87% der Patienten zuvor bereits eine Systemtherapie erhalten hatten.^28^ Die Ansprechraten von Cemiplimab beim metastasierten PEK waren deutlich höher als von anderen Systemtherapien. Migden et al. berichteten über die beste Gesamtansprechrate von 50% in der Phase‐I‐Kohorte und 48% in der Phase‐II‐Kohorte. 7% hatten eine komplette Remission. Das mediane krankheitsfreie Überleben und das Gesamtüberleben wurden zum Zeitpunkt der Datenauswertung nicht erreicht.^29^

Checkpoint‐Inhibitoren nach einer Organtransplantation können zu einer Abstoßungsreaktion führen. In der Übersichtsarbeit von Fisher et al. kam es bei 37% der Patienten, die mit Checkpoint‐Inhibitoren behandelt wurden zu einer Transplantatabstoßung und 14% starben an den Folgen.^30^ Für die Immuntherapie bei Patienten mit hämatologischen Malignomen, wie zum Beispiel chronischer lymphatischer Leukämie, liegen wenige Daten vor.^31^ Wir haben in unserer Klinik bei diesen Patienten niedrige Ansprechraten beobachtet.

In der Literatur wird ein schlechteres Ansprechen auf Cemiplimab von Patienten mit einem Primarius in nicht chronisch sonnenexponierten Hautarealen beschrieben. Die Kombination aus Cetuximab und 5‐FU ist eine Option für dieses Patientenklientel.^32^

In unserer Studienkohorte wurden bei 45% der Patienten unerwünschte Ereignisse beobachtet. Das akneiforme Exanthem war bei 8/20 Patienten die häufigste Nebenwirkung. Dereure et al. beschrieben eine akneiforme Dermatitis bei 57% der mit Cetuximab oder Cetuximab und Cisplatin behandelten Patienten.^33^ Häufige kutane Nebenwirkungen von EGFR‐Inhibitoren sind akneiforme Exantheme, Xerosis cutis und Paronychien, welche die Lebensqualität der Patienten erheblich beeinträchtigen können. Über 80% der mit EGFR‐Inhibitoren behandelten Patienten entwickeln papulopustulöse Läsionen im Gesicht oder am oberen Rumpf. Zu den Behandlungsmöglichkeiten gehören topische Akne‐ oder Rosazea‐Therapien, systemische Antibiotika (Doxycyclin 200 mg täglich oder Minocyclin 100 mg täglich) oder Isotretinoin (10–20 mg täglich).^34^, ^35^ In ihrer prospektiven Studie an 36 Patienten mit fortgeschrittenem PEK, die Cetuximab erhielten, berichteten Maubec et al. über Infektionen (22%) und Blutungen (11%) als häufigste Nebenwirkungen.^25^

In unserer Studie musste ein Patient die Behandlung aufgrund von Myelosuppression abbrechen. Die Myelosuppression ist neben Diarrhoe und Mukositis eine der häufigsten Nebenwirkungen der 5‐FU‐Chemotherapie.^36^ Kardiotoxizität von 5‐FU und Capecitabin wurde in der Literatur ebenfalls beschrieben. Leichte Symptome wie Brustschmerzen oder Hypotonie treten bei 0%–20% der Patienten auf, während eine schwere symptomatische Kardiotoxizität mit kardiogenem Schock bei ≤ 2% während der Fluorouracil‐Behandlung auftritt.^37^ Bei unseren Patienten wurden keine kardiovaskulären Ereignisse dokumentiert.

Eine Limitation dieser Studie ist das retrospektive Studiendesign und die geringe Patientenzahl. Darüber hinaus konnte das krankheitsspezifische Überleben nicht für alle Patienten genau bestimmt werden. Angaben zur Todesursache fehlten häufig, da die Patienten außerhalb unserer Klinik starben oder die genaue Todesursache nicht dokumentiert war. Außerdem waren die Patienten häufig multimorbide, was die Bestimmung der genauen Todesursache erschwerte. Daher wurde lediglich das Gesamtüberleben und nicht das krankheitsspezifische Überleben berechnet.

### Fazit

In unserer Patientenkohorte führte die Kombination von 5‐FU und Cetuximab bei 50% unserer Patienten zu einem partiellen Ansprechen oder einer stabilen Erkrankung. Das mediane progressionsfreie Überleben lag bei drei Monaten (95%‐KI 2–nicht erreicht) und das mediane Gesamtüberleben bei 29 Monaten (95%‐KI 11–nicht erreicht). Die häufigste Nebenwirkung war ein akneiformes Exanthem bei 40% der Patienten.

Die Kombination von Cetuximab und 5‐FU ist eine gut verträgliche Behandlungsoption für Patienten mit fortgeschrittenem kutanen PEK, bei denen eine systemische Erstlinientherapie mit Cemiplimab kontraindiziert ist oder es zu einer Progression des Tumors unter Anti‐PD1‐Therapie gekommen ist. Die Patienten können von dieser Kombination, die aufgrund ihrer geringen Toxizität auch für ältere Patienten mit Begleiterkrankungen eine Option darstellt, profitieren. Die Krankheitskontrollrate lag in unserer Kohorte bei 50%. Die Ansprechrate betrug 5%. Dies zeigt, dass weitere randomisierte prospektive Daten zu systemischen Behandlungsoptionen für Patienten mit fortgeschrittenem kutanen PEK, die für eine Anti‐PD1‐Therapie nicht in Frage kommen, erforderlich sind.

## DANKSAGUNG

Wir danken Fenja Grimm von der Zytostatika‐Apotheke der Charité Berlin für ihre Unterstützung bei der Datenerhebung und Dr. med. Vincent Walter für seine Unterstützung bei der Datenanalyse.

Open access Veröffentlichung ermöglicht und organisiert durch Projekt DEAL.

## INTERESSENKONFLIKT

Keiner.
